# Are Polymeric Membranes Truly Sustainable? Life Cycle Assessment Studies of Polymeric Membranes in Post-Combustion CO_2_ Capture: A Systematic Review

**DOI:** 10.3390/polym18070868

**Published:** 2026-04-01

**Authors:** Talha Kemal Koçak, Aytac Perihan Akan, Eric Favre

**Affiliations:** 1 Department of Environmental Engineering, Hacettepe University, Ankara 06800, Türkiye; talhakemalkocak26@hacettepe.edu.tr; 2 CNRS, LRGP, Université de Lorraine, F-54000 Nancy, France; eric.favre@univ-lorraine.fr

**Keywords:** gas separation, environmental impact, sustainable decarbonization, bio-polymers, green solvents, energy policy

## Abstract

Polymeric membranes are promising materials for post-combustion CO_2_ capture (PCC), yet their life cycle environmental performance remains uncertain. This review synthesizes 21 life cycle assessment (LCA) studies of polymeric membrane-based PCC systems to examine methodological choices, quantify environmental trade-offs, and identify research gaps. Google Scholar, Web of Science, ScienceDirect, and MDPI were searched up to January 2026. Methodological quality and risk of bias were assessed against a 10-criteria framework derived from ISO 14044. Results indicate widely varying system boundaries and functional units, with only four studies performing formal uncertainty analysis. Within individual study contexts, polymeric membrane gas separation systems can reduce global warming potential (GWP) by up to 89% compared to no-capture plants, though other impacts, like ozone depletion potential, increase by up to 780%. Compared to amine-based absorption, membranes showed superior performance, with reductions up to 26% in GWP and 98% in other categories. In some cases, large relative reductions are driven by scenario-specific baselines and should be interpreted with caution. Outcomes were most sensitive to background energy mixes and raw material demand. The absence of commercial-scale data highlights the need for harmonized frameworks and standardized functional units. Future research should prioritize membrane material selection, renewable energy integration, and coordinated policy–industry collaboration.

## 1. Introduction

As the main anthropogenic driver of global temperature rise, atmospheric CO_2_ has increased by about 50% compared to the preindustrial era [1]. This alarming increase has prompted international efforts to tackle climate change, with many countries setting ambitious goals to achieve net-zero CO_2_ emissions by 2050, including the United States, the European Union, China, and Japan [2]. To mitigate atmospheric CO_2_ levels, one short-term solution is the use of post-combustion CO_2_ capture (PCC) technologies. Within these technologies, chemical absorption via amine wash remains the most mature PCC system and yields high removal efficiency by using an amine/water solution to absorb CO_2_ from flue gas [3]. In contrast, membrane gas separation and absorption systems have received considerable attention due to their lower environmental impacts, higher chemical and thermal resistance, and compact design [4]. Of the various membrane types, polymeric membranes are considered the most promising because their porous surface provides high CO_2_ permeability, their lightweight composition facilitates integration into existing plants, their corrosion resistance protects plant equipment, and their relatively low energy demand reduces operational costs and environmental impact [5]. To better contextualize this, Table 1 presents a comparison of polymeric and other membranes based on key performance indicators, including CO_2_ permeance, selectivity, thermal/chemical stability, and integration potential. Despite these advantages, a systematic synthesis of the life cycle environmental performance of polymeric membranes for PCC remains largely unexplored.

In polymeric membrane PCC systems, numerous factors influence process efficiency and environmental performance. In gas separation membranes, there is a well-known trade-off between CO_2_ permeability and CO_2_/N_2_ selectivity; therefore, different material choices can lead to varying environmental outcomes [6]. Moreover, the membranes used in gas separation generally require a larger contact area per unit volume than other PCC technologies, which increases raw material demand during membrane fabrication [7]. In membrane gas absorption systems, absorbent regeneration introduces an additional environmental burden [2]. For both gas separation and absorption systems, variations in baseline assumptions result in different performance and environmental scenarios. These variations include, but are not limited to, material requirements for membrane fabrication, differences in system efficiency, the integration of auxiliary technologies, and the type of power plant considered. Despite these differences, several studies have shown that under optimized operational conditions, polymeric membrane-based CO_2_ capture systems can achieve significant reductions in global warming potential (GWP) (e.g., [7,8]). However, reductions in GWP alone are insufficient to fully assess the sustainability of polymeric membranes, as their impacts on other environmental categories must also be considered.

**Table 1:** The comparison of polymeric, ceramic, zeolite, and mixed-matrix membranes in terms of CO_2_ permeability, selectivity, stability, and scalability.

Membrane Type	Typical CO_2_ Permeance (GPU) ^1^	Typical CO_2_/N_2_ Selectivity	Chemical/Thermal Stability	Integration & Scalability	References
Polymeric (e.g., Polaris^TM^, PolyActive™)	1000–2000	20–60	Moderate; prone to plasticization and aging	Excellent: lightweight, low cost, modular	[9,10]
Ceramic (non-zeolite, e.g., alumina, silica, titania)	500–3000	10–50	Excellent chemical and thermal stability (>400 °C)	Limited by brittleness and high fabrication cost	[4,11]
Graphene-based (2D membranes)	≈10,000	10–50	High thermal stability; scalability challenges	Emerging technology; lab-scale demonstrations	[12]
Zeolite (e.g., DDR, MFI, CHA frameworks)	2000–4000	30–100	Excellent thermal/chemical stability	High selectivity but costly, challenging scale-up	[10,13]
Mixed-Matrix Membranes (MMMs, e.g., polymer + zeolite/MOF)	500–3000	30–100	Improved vs. neat polymers; filler enhances stability	Good, but filler dispersion and compatibility issues	[14,15,16]
Other Emerging MMMs (e.g., ZIF-7/Pebax)	1000–4000	40–130	Better than polymer alone; stability enhanced by ZIF filler	Attractive for processing; scalable	[17,18]

^1^ Industrial performance and scalability are determined by permeance (GPU), which equals permeability divided by the selective layer thickness. 1 GPU corresponds to a membrane of 1 µm thickness and 1 Barrer permeability.

Several studies have assessed the life cycle impacts of polymeric membrane PCC systems across impact categories such as global warming, ozone formation, freshwater eutrophication, human toxicity, and acidification. To evaluate these impacts, life cycle assessment (LCA) is an effective tool; however, extensive data are required to produce representative results. As a result, no standardized approach exists among LCAs, as each study examines distinct plant configurations and alternative scenarios. Moreover, LCA investigations differ in their objectives, leading to the adoption of varying functional units (FUs) and system boundaries. A gate-to-gate boundary excludes the extraction and production of membrane materials, whereas a cradle-to-gate boundary does not consider use-phase and end-of-life stages in environmental impact assessments. Consequently, the existing literature presents complex and sometimes conflicting findings regarding the environmental performance of polymeric membranes. For instance, Wang et al. [19] compared monoethanolamine (MEA)-based chemical absorption with two-stage membrane gas separation in a coal-fired power plant using a cradle-to-gate boundary and reported that membrane gas separation outperformed MEA in all impact categories. In contrast, Zhang et al. [20] employed a cradle-to-grave boundary and found that membrane gas separation led to higher ozone formation, particulate matter formation, and terrestrial acidification than MEA-based absorption. These contrasting findings illustrate how methodological choices strongly influence LCA outcomes. Consequently, evaluating individual studies in isolation may obscure the broader environmental profile of polymeric membranes, highlighting the need for a comprehensive synthesis of the available evidence to clarify the current state-of-the-art.

The lack of a standardized approach in LCA investigations, along with diverse operational conditions, poses a challenge to understanding the full extent of the sustainability implications of polymeric membranes for PCC. Therefore, questions remain about how polymeric membranes perform environmentally compared to a no-capture scenario and other separation methods, and which parameters should be considered to improve the system’s sustainability. These questions require a systematic investigation of the available literature on LCAs of polymeric membranes, which, to our knowledge, has not been done before. To address this gap, the current study systematically reviews the LCAs focusing on the use of polymeric membranes for PCC. Specifically, the study synthesizes the goals, scopes, functional units, and system boundaries adopted across 21 LCA studies, highlighting similarities, differences, and methodological variations. It also identifies the polymer materials, solvents, system types (e.g., membrane gas separation/absorption, mixed-matrix membranes), and module configurations (e.g., hollow fiber, spiral wound) reported in the literature. Furthermore, several reviewed studies have evaluated the environmental impacts of polymeric membrane-based PCC systems by comparing them with base-case scenarios without PCC and/or with alternative capture technologies. Accordingly, this SLR assesses EI values based on the relative differences reported between these scenarios, enabling a consistent comparison across membrane gas separation systems. By outlining research gaps and emphasizing future directions, this study provides a synthesis that reveals the current state-of-the-art and sets a foundation for advancing sustainable membrane-based CO_2_ capture.

## 2. Methodology

In order to ensure methodological accuracy, transparency, systematization, and subjectivity of the systematic review, the PSALSAR framework (Protocol, Search, Appraisal, Synthesis, Analysis, and Report) was used in the study [21]. Several systematic investigations in the environmental sciences have successfully applied this framework to enhance the robustness and acceptability of their research (e.g., [22,23,24]). Subsequent subsections detail the PSALSAR steps applied in the current study.

### 2.1. Protocol

The protocol step defines the research scope to articulate the research questions that the review work aims to answer. In the current study, PECO (population, exposure, comparator, and outcome) concept was used to determine the research scope [25]. The PECO offers a structured approach to research problem formulation, enabling the systematic analysis of research questions through the lens of their constituent concepts. Table 2 lists the PECO concepts and their application in the current study. Accordingly, refined research questions based on the research scope are listed below.

1. What Is the State-of-the-Art for Polymeric Membrane Gas Separation and Absorption Systems for PCC?
2. What are the common LCA approaches in the reviewed studies? How do the selected parameters influence potential environmental outcomes? Such parameters include but not limited to the methodological approach, material choice, system boundaries, functional units, and data source.
3. How do polymeric membranes perform environmentally compared to alternative scenarios, including no capture system and other PCC systems?
4. How do the reviewed studies address the sensitivity and uncertainty of their data? Which processes influence environmental outcomes the most?
5. What are the existing research gaps in the current body of LCA literature on polymeric membranes for PCC? How should future research address these gaps?

**Table 2:** The application of the PECO concept to define the scope of the research.

Content	Application
Overall Research Question	Is the use of polymeric membranes for PCC truly sustainable in terms of environmental impacts? If so, which polymeric materials and membrane systems, as well as solvents, should be preferred over others in terms of environmental concerns?
Population	Public health and the environment
Exposure	The environmental impacts of polymeric membranes for PCC
Comparator	No CO_2_ capture system or alternative PCC systems
Outcome	Potential impacts on environmental impact categories from global warming to ozone depletion

### 2.2. Search

This step involves developing a search strategy to identify relevant studies that address the research questions defined in the protocol [21]. This involves selecting appropriate search terms and the databases to which they will be applied. For the current study, three core components of the research questions were used to define the search strings: (1) polymeric membranes, (2) life cycle assessments, and (3) carbon capture processes. Based on the scope of the research, included studies should contain all three components, so the “AND” Boolean operator was used between the core components. Each component has alternative search strings that represent it. For the life cycle assessment component, for example, the search strings “life cycle assessment,” “LCA,” “life cycle analysis,” “environmental impact assessment,” “life cycle inventory,” and “life cycle impact assessment” were used. The “OR” Boolean operator was used between these alternative search strings to ensure that articles containing any of them appear in the search results. Generic search strings such as “polymeric membrane” and “carbon dioxide capture” were preferred so as not to miss any relevant studies, and doing so did not result in an unmanageable sample size. Lastly, the search strings were applied to the whole article domain rather than just the title or abstract. The full list of search strings and the number of identified studies is provided in Table S1.

The search strings were applied to four international databases, which are Google Scholar, ScienceDirect, Web of Science, and the Multidisciplinary Digital Publishing Institute (MDPI) relevant to the study’s topic. As a result, a total of 967 records were retrieved from all databases. This number reflects the broad initial search results obtained using inclusive search strings and therefore includes many studies that do not meet the specific scope of this review. These records were subsequently subjected to a structured screening process in the appraisal step.

### 2.3. Appraisal

**Figure 1:**
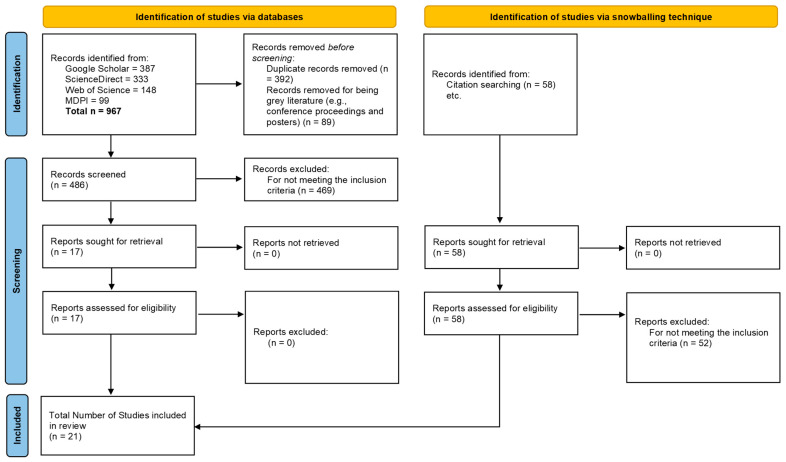
PRISMA 2020 flow diagram illustrating the systematic study selection process (n = 21), adapted from Page et al. [26].

In this step, the identified articles were evaluated to determine their inclusion status in the review (Figure 1). Inclusion criteria were defined based on the research scope and questions outlined in the protocol. The articles were included if they (1) focused on the life cycle assessment of polymeric membranes used for carbon dioxide capture in post-combustion processes, (2) included at least one polymeric component in the separation process, such as in mixed matrix or hybrid membranes, (3) were peer-reviewed journal articles, and (4) were written in English. To apply these inclusion criteria, the titles and abstracts of the 967 articles were read. Studies that did not meet these criteria were excluded from the review, as were duplicate articles. Following title and abstract screening, 17 studies were identified as meeting the eligibility criteria for full-text assessment. The substantial reduction from the initial records reflects the scope of this review, which specifically targets studies that simultaneously address the above 4 inclusion criteria.

While reading the full texts of the 17 articles, another technique called snowballing was applied to identify additional articles. This technique is effective for detecting articles that are not found using search strings alone, and it involves checking the references and citations of the selected articles [27]. Accordingly, the reference lists of the 17 articles were checked to determine if there were any relevant articles. For the citations of the 17 articles, the “Cited by” option on Google Scholar was used for each article. This process was repeated as needed for the newly identified articles. The application of snowballing identified 4 additional articles, bringing the total number of included studies to 21. The full list of included studies is given in Table S2.

#### Quality Assessment and Risk of Bias

To ensure the transparency and reliability of the synthesized evidence, the methodological quality of the included studies was systematically assessed. A critical appraisal framework was developed based on ISO 14044 standards [28,29], comprising ten key reporting and methodological criteria. These criteria evaluated the clarity of the LCA goal, functional unit, system boundaries, software utilization, data sources, assumptions, impact assessment methods, and the inclusion of sensitivity and quantitative uncertainty analyses. Each study was independently evaluated by two reviewers against these criteria and assigned a compliance score of ‘Yes’ (fully reported/conducted), ‘Partial’ (partially reported or lacking detail), or ‘No’ (not reported/conducted). Any discrepancies in scoring were resolved through consensus. The results of this assessment were used to characterize the robustness of the existing literature and to identify systemic research gaps in the field of polymeric membrane-based PCC systems.

### 2.4. Synthesis

In this step, relevant data was extracted from the selected articles to prepare them for analysis. The extraction process was conducted systemically using a matrix system on an excel sheet with each column representing a category. Table S3 depicts the selected categories, along with the corresponding contents and the rationale for extraction. The categories were determined based on the research scope and questions discussed in the protocol step (Section 2.1). Two reviewers independently read and coded every article, before comparing the results to confirm their relevance.

### 2.5. Analysis and Reporting

**Figure 2:**
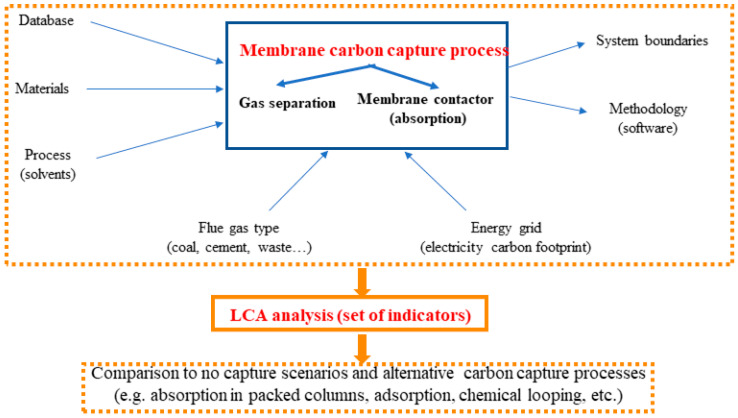
Conceptual framework of the reviewed LCA studies on polymeric membrane-based PCC systems.

During the analysis stage, the synthesized information was analyzed qualitatively and quantitatively to answer the research questions (Section 2.1). For the qualitative analysis, extracted data were reviewed and categorized without modification. This process involved identifying trends and patterns across the selected studies, including study objectives, system boundaries, FUs, LCA methodologies, and CO_2_ capture technologies (Figure 2). The analysis also considered the types of polymeric membranes studied, operational conditions, and target applications (e.g., coal-fired power plants, steel or cement production). Additionally, studies were compared in terms of their reported environmental impact categories, foreground and background data sources, and modeling approaches.

As for the quantitative analysis, we extracted environmental impact (EI) values from the reviewed studies. Due to substantial heterogeneity in system boundaries, functional units, and background assumptions across the reviewed studies, direct cross-study numerical comparisons of absolute environmental impact values are not methodologically valid. To address this limitation, this review adopts a within-study comparative approach, whereby environmental impacts of polymeric membrane systems are evaluated relative to benchmark scenarios (e.g., no-capture or amine-based systems) under consistent assumptions defined within each individual study. This approach minimizes methodological inconsistencies and allows for more reliable identification of directional trends (i.e., increases or decreases in impact categories), while avoiding misleading cross-study aggregation of absolute values.

Lastly, the PSALSAR framework’s reporting step requires a detailed description of the systematic review method and research findings [30]. To transparently report this study, PRISMA (Preferred Reporting Items for Systematic reviews and Meta-Analyses) guideline was followed [26]. PRISMA abstract and main checklists were provided in Supplementary Tables S4 and S5, respectively.

## 3. Results and Discussion

### 3.1. Study Quality and Reporting Transparency

**Figure 3:**
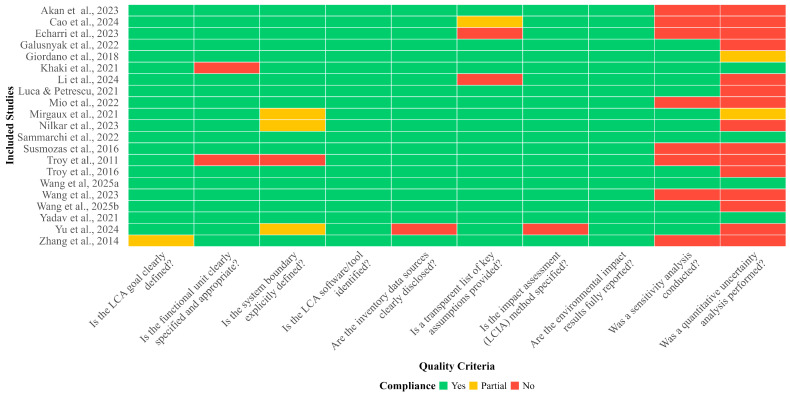
Methodological quality assessment and reporting transparency of the included LCA studies (n = 21). References include Akan et al., 2023 [1], Cao et al., 2024 [8], Echarri et al., 2023 [31], Galusnyak et al., 2022 [32], Giordano et al., 2018 [6], Khaki et al., 2021 [5], Li et al., 2024 [2], Luca & Petrescu, 2021 [7], Mio et al., 2022 [33], Mirgaux et al., 2021 [34], Nilkar et al., 2023 [35], Sammarchi et al., 2022 [36], Susmozas et al., 2016 [37], Troy et al., 2011 [3], Troy et al., 2016 [38], Wang et al, 2025a [39], Wang et al., 2023 [19], Wang et al., 2025b [40], Yadav et al., 2021 [41], Yu et al., 2024 [42], and Zhang et al., 2014 [20].

The systematic assessment of the 21 included studies revealed a high level of transparency regarding fundamental LCA components, yet significant gaps in advanced robust analyses (Figure 3). All studies (100%) clearly defined their LCA goals, and over 90% provided explicit functional units and system boundaries. Furthermore, inventory data sources and LCIA methods were well-disclosed across the majority of the papers. However, significant methodological risks were identified concerning the robustness of the reported environmental impacts. Specifically, only approximately 20% of the studies conducted a formal quantitative uncertainty analysis (e.g., Monte Carlo simulations), while sensitivity analysis was absent or only partially addressed in nearly half of the studies. This lack of rigorous uncertainty characterization suggests that while the environmental benefits of polymeric membranes are widely reported, the statistical confidence in these findings remains limited, underscoring the need for more harmonized and rigorous reporting standards in future membrane LCA research.

### 3.2. Polymeric Membranes in Post-Combustion CO_2_ Capture

Polymeric membranes encompass a diverse set of materials and configurations, ranging from conventional gas separation membranes to mixed-matrix membranes and membrane contactors used in absorption systems. The reviewed studies considered their use across different industrial contexts and scales, with varying degrees of technical maturity. To capture this diversity, we need to address their application context, operating principles and configurations, as well as the most commonly employed polymeric materials and solvents.

#### 3.2.1. Application Context

Polymeric membranes emerged in the 2010s as a candidate alternative to primary amine, particularly MEA scrubbing [43]. Since then, these materials have attracted considerable research interest because they can lower energy use and costs relative to conventional solvent systems, and they are straightforward to fabricate with good chemical resistance, thermal stability, and mechanical strength [5,44,45]. At the same time, they carry non-negligible life cycle burdens from manufacture through end-of-life, which has motivated a growing LCA literature [3,31]. Based on the reviewed studies, no LCA study has yet examined the commercial-scale application of polymeric membranes for PCC, with 76% of studies using theoretical process simulation/modeling, 14% relying on laboratory-scale experimental data, and 10% using the both (see Figure S1a). The most frequently examined contexts are coal-fired power plants (57%), membrane production facilities (19%), and steel mills (14%) (see Figure S1b). Given that power plants account for 60% of atmospheric CO_2_ emissions among major industrial sources, including refineries, cement plants, natural gas power plants, and steel and iron production facilities [46], this focus is reasonable.

#### 3.2.2. System Configurations and Operating Principles

The design and operation of polymeric membrane PCC systems largely depend on flue gas characteristics and required CO_2_ capture performance. In coal-fired power plants, flue gas typically contains CO_2_ (8–15% *v*/*v*), N_2_, O_2_, and trace components such as SO_x_ and NO_x_, which influence separation efficiency and system design. Polymeric gas separation membranes selectively allow CO_2_ to permeate through a thin film while retaining gases such as N_2_ and O_2_, based on molecular size, solubility, and diffusion coefficients [7]. Key performance targets include high CO_2_/N_2_ selectivity and high CO_2_ permeability, though attaining both simultaneously is challenging [6]. Membrane systems typically aim for capture rates above 80%, defined as the ratio of CO_2_ in the permeate to that in the feed. Achieving these rates often requires multi-stage configurations.

Accordingly, most reviewed studies examined two-stage (56%) or three-stage (14%) systems, followed by mixed-matrix (11%) and membrane gas absorption (8%) systems (Figure S1c). Regarding module configuration, 62% of scenarios did not specify the module type; among those reported, hollow-fiber membranes were most frequently applied (26%), likely due to their high surface area for mass transfer [47]. Spiral-wound membranes accounted for 12%, while other configurations such as flat-sheet and plate-and-frame modules remain underexplored.

Membrane systems operate in distinct configurations depending on separation strategy. In two-stage gas separation, CO_2_ permeates in the first unit and is further enriched in a second stage [33,40]. In membrane gas absorption systems, the membrane functions as a contactor separating gas and liquid phases, enabling CO_2_ diffusion into a liquid absorbent such as an amine or carbonate solution [1]. Mixed-matrix membranes enhance separation performance by incorporating inorganic particles into the polymer matrix [31]. There are also hybrid systems that integrate membranes with other PCC technologies, such as cryogenic separation or amine scrubbing, to reduce energy penalties and environmental impacts [1,20]. Across these configurations, the overarching objective is to increase CO_2_ capture efficiency while minimizing energy demand and environmental burdens.

**Figure 4:**
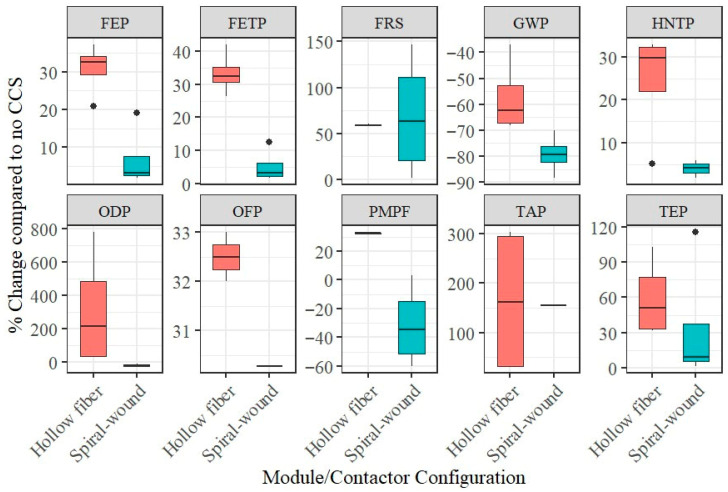
Relative environmental performance of Hollow-fiber and Spiral-wound membranes across impact categories, normalized to a reference case without CCS. Black dots denote outliers.

Available studies do not allow direct comparison of environmental performance across different membrane configurations. Only three studies have explored membrane gas absorption, and one has examined a membrane–cryogenic hybrid system -. In contrast, sixteen studies focus on membrane gas separation. Despite this limitation, available LCA scenarios for hollow-fiber (4) and spiral-wound (4) configurations allow comparison with a base case without carbon capture and storage (CCS) (Figure 4). Although spiral-wound modules showed lower impacts in the reviewed cases, both module types require comparable components (polymeric membranes, potting materials, and casings). Therefore, the observed differences are more plausibly attributed to variations in assumed material inventories and operating conditions than to intrinsic geometric advantages. For other impact categories, the sample size remains too small to draw meaningful conclusions.

#### 3.2.3. Polymeric Materials, Performance, and Solvent Choice

Polymer selection varied considerably across studies, with some employing multiple materials. Polysulfone was the most common (5 studies) as a selective membrane, followed by Polyvinyl amine (4) and PolyActive™ (4) (Figure S1d). Among the three membrane gas absorption applications, two used polypropylene as a membrane contractor, while the other did not report the contractor material.

Polyvinyl amine offers high CO_2_ permeance (0.28 m^3^ STP m^−2^ bar^−1^ h^−1^) under optimal feed pressure while maintaining CO_2_/N_2_ selectivity comparable to alternatives [7,32]. It can also serve as a coating for composite hollow-fiber membranes, improving permeability and selectivity [20]. Polysulfone provides balanced performance and durability and is widely used as a substrate for thin-film composites due to its thermal stability, mechanical strength, chemical resistance, and ease of fabrication [48]. However, its intrinsic CO_2_ permeability and selectivity remain lower than those of high-performance polymers [49]. PolyActive™ is a commercial block copolymer whose PEO/PBT ratio and PEO molecular weight determine separation properties. For example, PolyActive 1500 (77 wt% PEO) shows relatively high selectivity with moderate permeability [50], while low-molecular-weight PEG additives can increase CO_2_ permeability up to fivefold with minimal selectivity loss [51]. Polypropylene is widely used as a membrane contactor material due to its intrinsic hydrophobicity, chemical resistance to amine solvents, mechanical robustness, and low cost [52].

The reviewed studies employed these polymers either as single materials (e.g., polypropylene) or in combination (e.g., polysulfone/polyvinyl amine) (Table 3, Table 4 and Table 5). Across the 18 scenarios reporting environmental impact values, eight distinct polymer combinations were identified, limiting direct environmental comparison. Consequently, the available data are insufficient to determine which polymeric material performs best from an environmental perspective.

Of the 21 studies reviewed, only 5 reported the solvents used in membrane fabrication. Accordingly, Table 3 reflects only the materials explicitly reported in the reviewed LCA studies and does not represent the full range of polymeric membranes developed in the broader literature. Many solvents used in these studies fall into hazardous or problematic categories, which implies their potential contribution to environmental impact values across multiple impact categories. In contrast, Yadav et al. [41] and Echarri et al. [31] demonstrated that substituting fossil-based solvents with greener alternatives, such as ethylene carbonate or bio-based ionic liquids, can significantly reduce impacts such as GWP, fossil resource scarcity (FRS), human cancer toxicity potential (HCTP), and human non-cancer toxicity potential (HNTP). These findings indicate that solvent selection is a critical but often overlooked factor in the LCA of polymeric membranes for PCC, and future LCAs should systematically address this dimension to guide sustainable membrane fabrication. Taken together, both polymer composition and solvent selection seem to influence environmental performance of polymeric membrane PCC systems, but they remain inconsistently addressed across existing LCA studies.

### 3.3. Life Cycle Assessment of Polymeric Membranes for Post-Combustion CO_2_ Capture

For PCC technologies, it is important to evaluate not only technical performance but also the environmental and economic implications of membrane materials and operating conditions. While current commercial membrane applications focus predominantly on technical efficiency, environmental considerations are increasingly relevant in membrane selection and system design.

Although CO_2_ capture technologies are intended to reduce greenhouse gas (GHG) emissions, their overall environmental performance depends on the materials and energy required for production and operation, which may contribute to GHG emissions and other impact categories. Evaluating these trade-offs requires a comprehensive assessment, for which life cycle assessment (LCA) provides a suitable framework. LCA examines all stages of a product’s life cycle from raw material extraction and manufacturing to operation and end-of-life. It enables identification of emission hotspots and key parameters influencing environmental outcomes [1]. Thus, LCA supports decision-making by facilitating a balanced evaluation of process efficiency and environmental trade-offs.

Following ISO 14040 [28] and 14044 [29] standards, LCA is typically conducted in four stages: (1) defining the goal and scope, (2) creating a life cycle inventory (LCI), (3) performing a life cycle impact assessment (LCIA), and (4) interpreting the results [33,38]. The reviewed studies incorporated these stages with varying levels of detail.

#### 3.3.1. Goal and Scope

The goal and scope define the assessment’s purpose, FU, and system boundary. Across the 21 reviewed studies, goals ranged from comparing CO_2_ capture technologies (14 studies) to evaluating operational conditions (2 study), polymer manufacturing processes (4 studies), or biomass co-firing integration (1 study) (see Table 4 and Table 5).

The FU establishes a reference point that standardizes environmental impact (EI) values for comparison [32]. For example, when the FU is defined as 1 kWh of electricity generation, all impact categories, such as GWP, are quantified per 1 kWh, enabling consistent comparison of scenarios within the same system. The system boundary defines the extent of the assessment by specifying which stages are included, from raw material extraction to disposal. Depending on the study objective, different approaches are applied, such as gate-to-gate, cradle-to-gate, or cradle-to-grave [2]. While cradle-to-grave boundaries capture the full life cycle, more limited boundaries focus on specific stages. System boundary selection is therefore critical for ensuring consistency between study objectives and result interpretation.

Across the reviewed studies, FU and system boundary choices varied considerably, reflecting the diversity of PCC applications. For coal-fired power plants, FUs were typically defined as 1 kWh of electricity (7 studies) or 1 ton of CO_2_ captured (4 studies), whereas cement and steel sector-based studies employed 1 ton of product. System boundaries ranged from gate-to-gate (4 studies) to cradle-to-gate (11 studies) and cradle-to-grave (5 studies) with further variation in how “the gate” was defined, either at the capture process or at membrane material production. There was thus a structural heterogeneity across the reviewed studies.

#### 3.3.2. Life Cycle Inventory

The LCI stage involves compiling all relevant material and energy flows, as well as pollutant emissions, to support the subsequent LCIA [2]. Accurate LCI data are crucial because they directly determine the reliability and magnitude of impact results.

Across the reviewed studies, foreground data were primarily sourced from literature, industry reports, and open datasets (14 studies), frequently supplemented with process simulations to generate inventory inputs (Table 4 and Table 5). Five studies conducted laboratory experiments, simulating either flue gas and PCC systems or polymer production processes. Background data were mainly obtained from established LCI databases, including Ecoinvent (14 studies), GaBi (3 studies), and GREET (1 study), while three studies constructed background inventories based on literature and technical reports.

#### 3.3.3. Life Cycle Impact Assessment and Interpretation

In the LCIA stage, the outputs obtained from the LCI are linked to the corresponding environmental impact categories in order to estimate potential impacts [32]. This stage requires a tool that can quantify the potential environmental impacts of a process, service, or product. Of the twenty-one reviewed studies, ten employed ReCiPe, five used CML, and one applied both methods (Table 4 and Table 5). The remaining four studies used Ecoinvent, GREET, the European Platform, and GaBi, while one study did not state the tool used. Environmental impact categories covered a wide range, including global warming potential (GWP); particulate matter formation potential (PMFP); ozone formation and depletion potentials (OFP, ODP); human toxicity potentials, both carcinogenic and non-carcinogenic (HCTP, HNTP); water consumption potential (WCP); freshwater and marine eutrophication potentials (FEP, MEP); freshwater, marine, and terrestrial ecotoxicity potentials (FETP, METP, TEP); terrestrial acidification potential (TAP); and the scarcity of mineral and fossil resources (MRS, FRS).

The interpretation stage synthesizes the LCIA results and evaluates broader implications. The reviewed studies typically interpreted findings in their Discussion or Conclusion sections. Differences in goal, scope, FU, system boundaries, and data sources strongly affect LCA outcomes, necessitating cautious interpretation. Comparative assessments were thus generally conducted within each study, as cross-study numerical comparisons were unreliable due to methodological heterogeneity.

### 3.4. Environmental Impacts of Polymeric Membranes for Post-Combustion CO_2_ Capture

As discussed in Section 2.5 and Section 3.2, variations in LCA approaches across the reviewed studies impede meta-analysis and direct comparison of EI values for polymeric membranes in PCC applications. Additionally, only three studies investigated membrane gas absorption systems, and none reported relative impact comparisons, limiting the ability to assess their environmental performance. In contrast, several membrane gas separation studies evaluated EI values by comparing membrane-based PCC systems with base-case scenarios without PCC and/or alternative capture technologies. By synthesizing the relative differences reported in these comparisons, this section assesses the environmental performance of polymeric membrane gas separation systems within a consistent comparative framework.

#### 3.4.1. Comparison with Base-Case Systems Without CO_2_ Capture

GWP is a relative metric used in LCA and climate science to compare the radiative forcing of a greenhouse gas to that of CO_2_ over a defined time horizon (e.g., 20, 100, or 500 years) [54]. It expresses the potential contribution of 1 kg of a gas to global warming as CO_2_-equivalents (CO_2_-eq), with CO_2_ assigned a GWP of 1.

**Figure 5:**
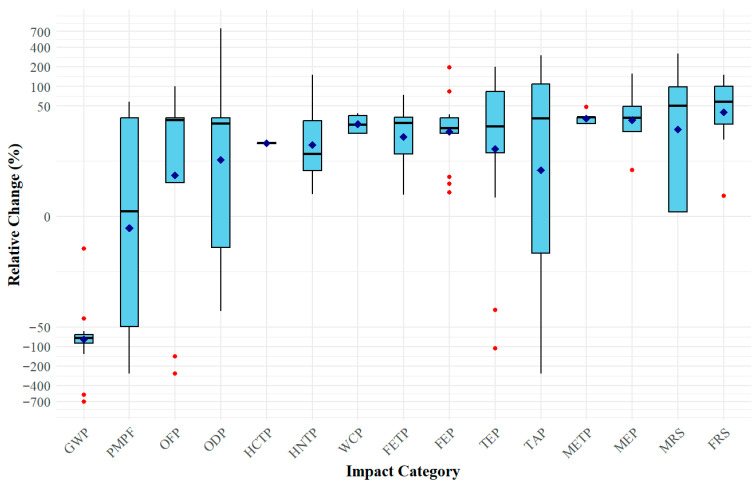
Box plots showing the relative percentage change in environmental impact categories for polymeric membrane gas separation systems used in post-combustion CO_2_ capture compared to a base-case without CO_2_ capture. Red dots and blue diamonds denote outliers and mean values, respectively.

According to the reviewed studies, GWP decreased by 3–705% in plants implementing polymeric membrane gas separation for PCC compared with base-case plants without PCC, with most results falling within the 60–90% range (Figure 5). These percentage reductions are highly dependent on baseline assumptions, capture efficiency, and system configuration, and therefore should not be interpreted as universally representative performance improvements. The dominant contributor to GWP was energy supply, as PCC systems require additional energy for operation [38]. When coal powered the capture system, upstream activities including mining, processing, and transport accounted for up to 90% of total GWP [2,19]. In contrast, CO_2_ transportation and storage contributed less than 10%, even for long-distance storage (e.g., 400 km) [32,38]. The smallest GWP reduction (3%) was reported by Mirgaux et al. [34], who applied a gate-to-gate boundary to a coal-fired power plant integrated with a chemical facility. The negligible improvement was primarily attributed to high energy demand and a low capture rate (≈2%). Conversely, the largest reduction (705%) was observed in a carbon capture and utilization (CCU) scenario. Yu et al. [42] evaluated several cradle-to-gate CCU scenarios in a steel mill and reported a 705% decrease in GWP for a system coupling polymeric membrane gas separation with methanol synthesis. This substantial reduction resulted from carbon recycling, as captured CO_2_ was converted into methanol rather than emitted. However, such extreme reductions are highly scenario-specific and reflect the inclusion of avoided emissions credited to downstream product utilization. As a result, these values are not directly comparable to conventional CCS systems, where CO_2_ is permanently stored rather than reused. Therefore, these large reductions represent upper-bound outcomes under specific system configurations, rather than representative performance of membrane-based CO_2_ capture technologies.

Despite GWP reductions, several impact categories increased relative to base-case plants (Figure 5). These increases were largely associated with fossil-based energy supply and material production. Coal combustion and upstream extraction release toxic substances (e.g., hydrogen fluoride, hydrogen chloride, phosphate, ammonia, chlorine, and NO_x_), elevating HCTP, HNTP, WCP, FETP, FEP, METP, MEP, MRS, and FRS [2,19,20]. Membrane fabrication also contributed to several impact categories. Production consumes mineral resources and typically involves organic solvents such as N-methyl-2-pyrrolidone (NMP), benzene, dimethylformamide (DMF), and dimethylacetamide (DMAc) [41]. Although effective for dissolving polymers during phase inversion and casting, these solvents are toxic and persistent, contributing to HCTP, HNTP, and OFP [5,41].

In certain scenarios, membrane-based PCC systems outperformed base-case plants in additional categories, including PMFP, TAP, ODP, OFP, and TEP (Figure 5). Cao et al. [8] reported that a two-stage membrane system reduced PMFP and TAP by 50% and 57%, respectively, primarily due to lower stack emissions of NO_x_, SO_2_, PM_2.5_, and NH_3_. Similarly, Wang et al. [39] observed a 78% reduction in TAP, linked to a 95% decrease in SO_2_ emissions. In a cement plant case, Galusnyak et al. [32] reported 20–60% reductions in ODP and PMFP using a spiral-wound membrane system powered by renewable electricity. Yu et al. [42] also found reductions in OFP and TEP in CCU scenarios involving methanol production or steel slag carbonization, attributable to CO_2_ recycling.

These findings suggest that polymeric membrane gas separation systems consistently reduce GWP relative to base-case plants without capture; however, increases in toxicity-, eutrophication-, and resource-related impact categories are frequently observed unless low-carbon energy supply or carbon utilization pathways are integrated into the system design.

#### 3.4.2. Comparison with Amine-Based Chemical Absorption

**Figure 6:**
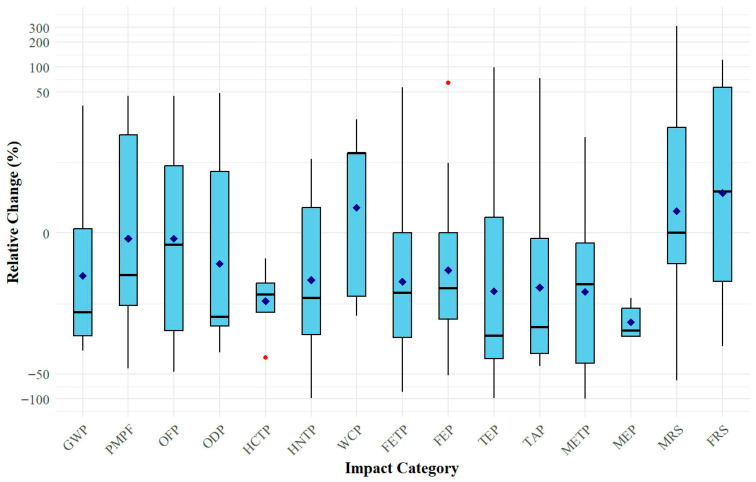
Box plots showing the relative percentage change in environmental impact categories for polymeric membrane gas separation systems used in post-combustion CO_2_ capture compared to amine-based chemical absorption systems. Red dots and blue diamonds donate outliers and mean values, respectively.

Chemical absorption by amine wash is the most mature and widely applied PCC technology in use today [34]. Of the twenty-one reviewed studies, fourteen compared amine-based absorption with polymeric membrane gas separation systems in terms of environmental impacts (EIs) (Table 4). Accordingly, membrane systems generally demonstrate favorable environmental performance relative to amine-based systems in several impact categories (Figure 6). In particular, substantial reductions (23–99%) were reported for GWP, ODP, HCTP, HNTP, FETP, FEP, TEP, TAP, and MEP. While these reductions indicate a general advantage of membranes systems over amine-based absorption, the magnitude of improvement varies across studies and is influenced by differences in process assumptions and energy integration. Nevertheless, the differences were primarily attributed to solvent regeneration and degradation in amine-based systems [6,20,32]. Solvent regeneration requires significant heat input, increasing energy demand and upstream coal-related emissions. In addition, amine degradation during operation releases toxic by-products that adversely affect human health and ecosystems [19,20]. By contrast, membrane gas separation systems avoid solvent regeneration and typically involve lower solvent use during fabrication, contributing to lower impact values in these categories [5,35].

In some cases, amine-based chemical absorption performed better, in certain impact categories (Figure 6). Luca and Petrescu [7] reported lower FRS, WCP, FETP, FEP, and OFP values for chemical absorption compared to membrane systems in a steel mill, likely due to the relatively high energy demand of O_2_ sequestration in the membrane configuration. Similarly, in a still mill, Mio et al. [33] observed higher WCP for membrane systems. Although the underlying cause for this was not explicitly stated, it may relate to differences in water management: amine systems often achieve efficient water recycling, whereas membrane processes can generate reject streams with elevated pollutant concentrations that limit water reuse [55,56]. Regarding resource scarcity, higher MRS values for membrane systems may stem from greater metal requirements during module construction and maintenance compared to absorption units [19,38]. The comparative evidence indicates that membrane gas separation systems demonstrate broader environmental advantages than amine-based absorption, although a few impact categories remain context-dependent.

#### 3.4.3. Comparison with Alternative CO_2_ Capture Technologies

Beyond amine-based absorption, several studies have compared polymeric membrane PCC systems with alternative capture technologies, including cryogenic and ceramic membrane separations for oxyfuel process; Selexol scrubbing for pre-combustion capture; and calcium looping, pressure swing adsorption (PSA), and temperature swing adsorption (TSA) for PCC. Troy et al. [38] compared membrane, absorption, oxyfuel (cryogenic and ceramic), and pre-combustion (Selexol) systems. They concluded that an integrated gasification combined cycle (IGCC) with Selexol exhibited the most favorable environmental profile because of its assumed high efficiency. However, they noted that this configuration has not yet achieved commercial maturity, and its projected performance remains uncertain.

In a coal-fired power plant, Zhang et al. [20] reported that a hybrid membrane–cryogenic system outperformed a two-stage membrane configuration across all impact categories, primarily due to lower energy demand. Similarly, Cao et al. [8] compared membrane gas separation, chemical absorption (MEA and ammonia), adsorption (PSA and TSA), and calcium looping in a coal-fired plant. Calcium looping demonstrated superior performance in several categories, largely because heat released during carbonation was assumed to be recovered for electricity generation. A comparable trend was observed in a cement plant study by Galusnyak et al. [32], where calcium looping again outperformed membrane and absorption systems due to favorable energy integration. In contrast, Yu et al. [42] reported that TSA with activated carbon achieved the lowest impacts in a steel mill context, outperforming membrane and absorption systems through an efficient methanol synthesis pathway. A broader environmental and techno-economic assessment by Wang et al. [39] found that although calcium looping achieved the best environmental scores, membrane gas separation offered a more balanced trade-off, combining moderate environmental impacts with life-cycle cost reductions of up to 70%. In another coal-fired power plant assessment, Mirgaux et al. [34] observed minimal differences between membrane separation and TSA, as both exhibited comparable energy requirements.

This synthesis indicates that while calcium looping and adsorption-based systems often achieve lower impact values under favorable integration assumptions, polymeric membrane systems remain competitive—particularly when environmental and economic considerations are evaluated jointly. Their simple design, modularity, and cost-reduction potential make them a viable alternative in contexts where environmental performance is not the sole criterion for decision-making.

#### 3.4.4. Sensitivity and Robustness Assessment of Comparative Environmental Impact Results

To evaluate the robustness of the synthesized EI trends, a “leave-one-out” sensitivity analysis was performed. Given that the EIs were evaluated as relative percentage changes within individual studies, this analysis involved sequentially excluding identified outliers and studies characterized by a high risk of bias—specifically those lacking transparency in LCA elements and uncertainty analysis. By recalculating the mean, maximum, and minimum relative changes for each iteration, we ensured that the comparative syntheses (Figure 5 and Figure 6) were not disproportionately influenced by anomalous data points.

The results indicate that the comparison between polymeric membranes and amine-based absorption is highly robust. Even when removing studies with the most extreme reductions or high risk of bias, membranes consistently maintained their superior environmental performance across all categories. While the magnitude of these advantages fluctuated with relative change reaching up to 226%, the directional trend remained stable, confirming the environmental competitiveness of membranes over chemical absorption. In contrast, the comparison against the no-capture base case exhibited significant sensitivity to specific methodological choices. Notably, the exclusion of the CCU scenario by Yu et al. [42] fundamentally altered the trends for OFP and PMPF. Because this specific CCU context yielded extreme reductions, its removal reversed the apparent advantages of membranes in these categories, resulting in a net increase in OFP (40%) and PMPF (1%) relative to the base case.

Despite these localized sensitivities, the core conclusions of the review remain valid: while polymeric membrane-based PCC systems offer a clear environmental advantage over conventional amine systems, they generally increase the majority of EI categories compared to a no-capture scenario, with the notable exception of GWP.

#### 3.4.5. Evaluating the Quality and Comparability of LCA Evidence

When comparing polymeric membrane-based CO_2_ capture systems across LCA studies, findings must be interpreted cautiously due to methodological inconsistencies and data limitations. As discussed in Section 3.3, life cycle results are strongly influenced by study-specific choices, including goal and scope, system boundaries, functional units, and data sources. These differences complicate cross-study comparability and may obscure which capture technology performs best under realistic conditions. To reduce bias from divergent assumptions, EI comparisons in this review were conducted within individual studies. Nevertheless, the sensitivity and uncertainty embedded in the underlying LCAs warrant closer examination.

A major source of uncertainty is the limited large-scale deployment of polymeric membranes and several alternative PCC systems. Most LCAs rely on simulations, pilot-scale data, or extrapolated assumptions rather than validated industrial performance. Consequently, assumptions regarding system efficiency, membrane lifetime, heat integration, and material requirements substantially influence outcomes. For example, studies reporting superior performance of calcium looping often assume optimistic heat recovery during carbonation (e.g., [32,39])—an option not equally available to membrane or absorption systems. Because energy demand is typically the dominant contributor to GWP and several other impact categories, such assumptions can significantly alter comparative results.

System boundaries are another critical driver of variation. Of the 21 studies reviewed, only 5 adopted a cradle-to-grave boundary, excluding upstream processes (e.g., raw material extraction and solvent or polymer production) and downstream stages (e.g., transport, storage, and end-of-life). Nevertheless, omitting capture material production can be misleading when quantifying the EI values, as polymer and sorbent synthesis can represent a substantial share of emissions and resource use.

Process design and material parameters also affect EI values across the process chain. Giordana et al. [6] showed that higher-selectivity membranes reduce power demand and improve GWP, TAP, TEP, and OFP, although larger membrane areas increase material burdens. Thus, they proposed a two-stage configuration as a balanced solution. Mirgaux et al. [34] found that reducing membrane thickness by 97% lowers ODP and HNTP by about 5%, but decreases CO_2_ purity by 11%. Luca and Petrescu [7] demonstrated that upstream chemical production routes (e.g., benzene, chlorine, sodium hydroxide) significantly influence LCA outcomes. Similarly, Khaki et al. [5] reported that a 20% change in dimethylformamide (DMF) consumption alters impact scores by 14–20%, particularly affecting ODP, HNTP, and FEP. Across the broader system boundary, energy source and raw material demand consistently emerge as the most influential parameters: Wang et al. [39] identified coal consumption as the dominant driver in coal-based PCC systems, while Nilkar et al. [35] highlighted electricity use and membrane material demand. Troy et al. [38] further emphasized the role of upstream factors such as coal type, mining location, and membrane lifetime. These findings collectively indicate that membrane manufacturing assumptions and energy-related parameters represent critical sources of variability. And given that these factors both dominate environmental burdens and exhibit high uncertainty, rigorous sensitivity and uncertainty analyses are essential for credible findings.

The LCA evidence on polymeric membrane PCC systems is highly sensitive to energy mixes, material choices, and methodological boundaries. Yet, uncertainty analysis remains underdeveloped. Of the twenty-one reviewed studies, only four conducted formal uncertainty analyses, compared to thirteen that included sensitivity tests.

The limited application of formal uncertainty analysis introduces an additional layer of interpretative uncertainty in this review. Because most studies rely on deterministic point estimates derived from simulation-based inventories, the reported EIs may not adequately reflect the variability associated with key parameters such as energy consumption, membrane lifetime, and material inputs. As these parameters are consistently identified as dominant contributors to EIs, their uncertainty may significantly affect both the magnitude and, in some cases, the direction of reported results. This has important implications for the confidence of the synthesized findings. The conclusion that polymeric membrane systems consistently reduce GWP relative to base-case scenarios is considered relatively robust, as this trend is observed across multiple studies despite methodological differences. In contrast, conclusions regarding trade-offs in other impact categories (e.g., toxicity, eutrophication, and resource depletion) are subject to greater uncertainty, as these categories are more sensitive to assumptions related to material production, solvent use, and background energy systems. Therefore, the lack of rigorous uncertainty characterization in the underlying literature reduces the confidence in the precision of reported environmental trade-offs, although the general directional trends identified in this review remain informative.

Based on these constraints regarding sensitivity and uncertainty of LCA evidence, Future research should prioritize (I) transparent reporting of assumptions, (II) standardized boundary definitions, (III) integration of pilot-scale or demonstration data, and (IV) systematic application of uncertainty and sensitivity analysis. For policymakers and industry stakeholders, these findings imply that while membranes hold promise as a low-impact CO_2_ capture option, investment and deployment decisions must be made cautiously, grounded in scenarios that reflect realistic operating conditions with validated data.

## 4. Limitations

This review has certain limitations that should be acknowledged. First, the analysis only included articles written in English, which may have excluded insights from studies published in other languages. At the same time, focusing on English-language publications ensured access to a broad set of internationally recognized studies, improving comparability and accessibility. Second, the review is limited to the scope of databases and search strategies applied, and some studies may have been overlooked. Even so, the PSALSAR approach and the inclusion of diverse journals and interdisciplinary sources strengthen the representativeness of the sample. Finally, while the review identifies key methodological and industrial gaps in the literature, it does not quantitatively assess their relative importance, as the heterogeneity of functional units, system boundaries, and methodological choices across the studies prevents a consistent weighting of these gaps. On the other hand, the qualitative synthesis highlights recurring issues, such as inconsistent system boundaries or overlooked impact categories, which can guide more focused and quantitative work in future reviews or meta-analyses.

## 5. Recommendations and Future Research Directions

Polymeric membranes for post-combustion CO_2_ capture present significant opportunities, but also substantial technical, environmental, and methodological challenges. A primary research priority is the transition from laboratory- and simulation-based studies to pilot- and commercial-scale demonstrations, which are essential to validate environmental and operational performance under realistic conditions. Even at commercial scale, however, reducing overall environmental burdens remains complex. The reviewed studies indicate that while membrane-based PCC systems generally reduce GWP in industrial applications, they often increase other impact categories such as OFP, TEP, FRS, and HNTP (Figure 5). These burden-shifting trade-offs are frequently underemphasized yet may offset climate benefits depending on regional context. For example, a membrane PCC system may reduce GWP but exacerbate water scarcity or health risks if deployed in water-stressed regions with vulnerable populations.

To address these challenges systematically, future research directions can be grouped into four interrelated domains: (i) material and manufacturing innovations, (ii) process and energy system integration, (iii) economic feasibility and competitiveness, and (iv) methodological standardization and policy support.

### 5.1. Material and Manufacturing Improvements

Reducing environmental impacts requires intervention across the entire membrane production chain. One of the most impactful strategies is substituting toxic, fossil-derived solvents with greener alternatives or implementing solvent recycling and recovery systems. This approach aligns with circular economy principles and can substantially reduce human toxicity, marine ecotoxicity, and energy demand [57]. Green solvents are typically biodegradable, renewable, and characterized by low volatility and toxicity [58]. They include ionic liquids, bio-sourced solvents, deep eutectic solvents (DES), supercritical fluids, green synthetic solvents, and water. These alternatives have been successfully applied in membrane fabrication, replacing conventional solvents such as N-methyl-2-pyrrolidone (NMP) and dimethylformamide (DMF). Importantly, several studies demonstrate that membranes fabricated using green solvents can achieve comparable or improved CO_2_/N_2_ selectivity while reducing solvent-related burdens (e.g., [16,59]).

Recent research emphasizes the potential of DES and ionic liquids not only as greener solvents but also as functional additives in mixed-matrix membranes. For instance, incorporating a choline chloride (ChCl)-based DES with glycerol into PES/SAPO-34 membranes significantly increased CO_2_ permeability while maintaining CO_2_/N_2_ selectivity [60]. The process was also promising in terms of CO_2_/CH_4_ separation performance. Moreover, DES are increasingly viewed as cost-effective and environmentally preferable substitutes that can enhance CO_2_ affinity [61]. Expanding the adoption of such solvent systems offers dual benefits: toxicity reduction and performance enhancement.

Another promising direction is substituting fossil-based polymers (e.g., PVDF) with bio-based alternatives such as cellulose acetate (CA). Preliminary findings suggest CA may modestly reduce GWP and resource depletion, particularly when greener acetylation routes and alternative biomass feedstocks are employed [41]. Additional candidates—including polylactic acid (PLA), chitosan, and polyhydroxyalkanoates (PHA)—are renewable, biodegradable, and compatible with green solvent systems [62]. Starch- and lignin-derived polymers also show promise when chemically modified to enhance durability. Diversifying polymer feedstocks could therefore reduce fossil dependency while maintaining favorable permeability–selectivity trade-offs.

In addition to conventional polymeric membranes, recent developments in polymer inclusion membranes (PIMs) highlight a promising direction for more sustainable membrane fabrication. PIMs typically consist of a polymer matrix incorporating carriers and plasticizers, enabling selective transport while potentially reducing the need for hazardous solvents and energy-intensive processing steps. Recent studies emphasize the use of greener components, such as bio-based polymers and low-toxicity additives, to improve both separation performance and environmental compatibility [63]. Despite these advantages, PIMs remain largely absent from current LCA literature on post-combustion CO_2_ capture. Their integration into future life cycle assessments could provide valuable insights into whether these emerging materials can reduce the environmental burdens associated with membrane fabrication, particularly in toxicity- and solvent-related impact categories.

### 5.2. Process and Energy System Integration

Beyond materials, fabrication and operational energy choices are critical leverage points. Transitioning from fossil-based electricity to renewable sources during polymer casting and curing can substantially lower GWP, acidification, and toxicity potentials [31].

At the operational level, hybrid membrane systems may mitigate acidification and toxicity burdens if designed with integrated solvent recovery, water recycling, and flue gas pretreatment units [20]. Downstream integration provides additional improvement pathways: waste heat recovery can reduce energy penalties; renewable power or biomass co-firing can limit net emissions; and utilization of captured CO_2_ (e.g., as chemical feedstock or in enhanced oil recovery) may enhance resource efficiency and reduce reliance on geological storage [36,64]. Solar-assisted systems have also demonstrated potential to lower impacts across multiple categories, including global warming, acidification, eutrophication, and resource depletion [40]. Future studies should evaluate these integration scenarios holistically, considering both process efficiency and multi-category environmental impacts.

### 5.3. Economic Feasibility and Competitiveness

While green solvents, bio-based polymers, renewable electricity, and hybrid systems are promising, their environmental advantages must be balanced against cost, scalability, and operational performance. Without techno-economic competitiveness relative to conventional fossil-based systems, large-scale adoption will remain limited.

A critical limitation across the reviewed literature is the lack of validation at commercial scale. Most studies rely on process simulations or laboratory-scale data, which may not fully capture the operational complexities encountered in industrial applications. In particular, membrane lifetime, fouling behavior, and long-term stability remain insufficiently characterized, despite their direct influence on both environmental impacts and economic viability. Furthermore, large-scale deployment requires integration with existing industrial infrastructure, which may introduce additional energy penalties, material demands, and system inefficiencies not reflected in idealized modeling scenarios. As a result, environmental benefits reported in the literature may not be fully realized under real operating conditions.

Similar challenges have been identified in broader environmental and techno-economic assessments of carbon capture systems, where environmental benefits observed at the laboratory or pilot scale may not directly translate to large-scale deployment due to economic constraints, process inefficiencies, and system integration challenges [65]. This finding highlights the importance of jointly evaluating environmental performance, scalability, and economic feasibility when assessing emerging technologies such as membrane-based CO_2_ capture.

Therefore, current LCA findings should be interpreted as indicative of technical potential rather than confirmed large-scale performance. Bridging this gap will require pilot- and commercial-scale studies that integrate process performance, durability, and economic constraints into LCAs. Accordingly, future research should increasingly combine environmental and techno-economic modeling to ensure realistic feasibility assessments.

### 5.4. Methodological Standardization and Policy Support

At the methodological level, improving comparability and robustness in LCA research is essential. Harmonizing functional units, system boundaries, and impact categories would significantly enhance cross-study comparability. Sensitivity and uncertainty analyses should be systematically applied—particularly to membrane lifetime, energy consumption, and material demand, which consistently dominate environmental outcomes.

LCA frameworks should also evolve beyond environmental assessment to incorporate economic and social dimensions, enabling full life cycle sustainability assessments. Furthermore, industrial deployment will require attention to recycling strategies and material circularity, given the substantial use of metals and solvents in membrane production. Finally, policy incentives and stronger academia–industry collaboration will be critical to accelerating the transition from promising laboratory concepts to commercially viable membrane technologies.

## 6. Conclusions

A systematic review of LCA studies on polymeric membrane-based PCC was conducted to identify environmental trade-offs, methodological choices, and opportunities for future research. The findings indicate that membrane systems can offer notable reductions in GWP compared to no-capture and chemical absorption scenarios; however, these benefits are highly dependent on energy sources, system configurations, and modeling assumptions. In particular, the results demonstrate that improvements in climate change impacts are often accompanied by increases in other categories compared to no-capture scenarios, such as toxicity, resource depletion, and ozone depletion. These environmental trade-offs point to the importance of energy sources, material selections, and process innovations.

This study establishes a foundation for sustainable polymeric membrane-based CO_2_ capture by synthesizing the current state-of-the-art. Specifically, it emphasizes the development of bio-based polymers, the adoption of green solvents, a greater reliance on renewable energy, and the inclusion of more commercial-scale assessments. Key challenges remain in (1) translating laboratory- and model-based membrane systems to industrial-scale applications, (2) reducing energy demands through efficiency improvements and renewable integration, and (3) advancing sustainable polymer and solvent synthesis. Without progress in these areas, the sustainability advantage of polymeric membranes compared to alternative capture scenarios may remain uncertain. Ultimately, polymeric membranes represent a credible pathway toward CO_2_ mitigation, but their success largely depends on integrating material innovations and cleaner energy choices, ensuring that gains in GWP are not offset by increases in other environmental burdens.

## Figures and Tables

**Table 3:** Polymer types and solvent systems reported in the reviewed LCA studies of polymeric membrane-based PCCs with the corresponding toxicity classification of Prat et al. [53].

Reference	Polymer(s)	Solvents Used for Membrane Manufacture	Prat et al. [53] Classification
[19]	Polysulfone	2,4-dichlorophenol, benzene, bisphenol (for polysulfone synthesis); ethylene carbonate (green solvent) for hollow fiber membrane production	Benzene = highly hazardous; ethylene carbonate = recommended (green solvent); 2,4-dichlorophenol = hazardous
[31]	Chitosan	Water, ethanol, formaldehyde, methylamine for ionic liquid (green solvent); deionized water with HCl for chitosan synthesis; water, formaldehyde, methanol, methylamine, benzene, ethanol, and toluene for different fillers	Water = preferred; ethanol = recommended; formaldehyde = hazardous; methylamine = hazardous; Methanol = recommended (low hazard); Benzene = highly hazardous; Toluene = problematic
[7]	Polysulfone/Polyvinyl amine	PVAm from polyacrylamide (PAA), NaOCl, NaOH; polysulfone from bisphenol A sodium salt and 4,4-dichlorodiphenyl sulfone; 4,4-dichlorodiphenyl sulfone from chlorobenzene and SO_3_; chlorobenzene from benzene and chlorine	Chlorobenzene = problematic (precursor); Benzene = highly hazardous; chlorobenzene = problematic. Others not classified
[5]	PAN, PVIM, PAN-co-VIM	DMF, water, hexane, methanol, acetone	DMF = hazardous; hexane = hazardous; acetone = recommended; methanol = recommended; water = preferred
[41]	Polyvinylidene, Polysulfone, Cellulose acetate	NMP, DMAc, DMF (fossil-based solvents); ethylene carbonate (green solvent)	NMP = problematic; DMAc = hazardous; DMF = hazardous; ethylene carbonate = recommended (green solvent)

**Table 4:** Overview of reviewed studies on polymeric membrane gas separation for PCC systems, detailing the plant type, LCA goals, functional units, system boundaries, data sources, and LCA tools and methods.

Selective Polymer(s) Studied	Configuration	Feed/Flue Gas Composition ^1^	Plant (Approach)	Goal/PCC Systems Studied	FU	System Boundary	Foreground/Background Data Sources	LCA Tool/EIA Method	Ref.
Polysulfone	Hollow-fiber membrane	Na ^2^	Coal-fired Power Plant (Theoretical)	EI comparison/Two-stage membrane, MEA absorption	1 kWh of electricity generation	Cradle-to-gate	Literature, industry reports, open data sets/Ecoinvent	OpenLCA/ReCipe	[19]
PolyActive^TM^	Na	Na	Biohydrogen production plant (Theoretical)	EI comparison/biohydrogen with CO_2_ capture	1 kg of hydrogen production	Cradle-to-gate	Literature/Ecoinvent	SimaPro/ IPCC, VDI, CML	[37]
Polyvinyl amine/ Polyphenylene oxide	Hollow-fiber membrane	3.65% O_2_, 13.73% CO_2_, 72.86% N_2_, 85 mg/Nm^3^ SO_2_, and 120 mg/Nm^3^ NO_x_	Coal-fired Power Plant (Theoretical)	EI comparison/Two-stage membrane, membrane-cryogenic hybrid, MEA absorption	1 kWh of electricity generation	Cradle-to-grave	Literature, industry reports/Ecoinvent	Ecoinvent/ReCipe	[20]
Chitosan	Na	Na	Polymer manufacture (Experimental and Theoretical)	EI evaluation/Membrane fabrication of chitosan-based MMMs	Permeate flow rate of 2.11 × 10^−3^ cm^3^ (STP)/s CO_2_	Cradle-to-gate	Literature, experimental/GaBi	GaBi/European Platform on LCA	[31]
PIM-1, PolyActive	Na	2.4% O_2_, 13.5% CO_2_, 68.1% N_2_, 15.2% H_2_O, and 0.8% Ar	Coal-fired Power Plant (Theoretical)	EI comparison/two-stage membrane, MEA absorption	1 ton of CO_2_ capture	Cradle-to-grave	Literature/Ecoinvent	SimaPro/CML	[6]
PolyActive^TM^	Na	Na	Coal-fired Power Plant (Theoretical)	EI comparison/Two-stage membrane, MEA absorption, cryogenic separation, ceramic membrane	1 kWh of electricity generation	Cradle-to-grave	Literature/Ecoinvent	GaBi/ReCipe	[38]
Polyvinyl amine	Na	Na	Steel Mill (Theoretical)	EI comparison/Two-stage membrane, MEA absorption	1 ton of steel production	Cradle-to-gate	Literature/Literature, GaBi	GaBi/ReCipe	[7]
Na	Na	Na	Coal-fired Power Plant (Theoretical)	EI comparison/Two-stage membrane, MEA and ammonia absorptions, calcium looping, pressure swing and temperature swing adsorptions	1 kWh of electricity generation	Cradle-to-grave	Literature, Industry reports/Literature	SimaPro/ReCipe	[8]
Polysulfone/Polyvinyl amine	Spiral-wound membrane	6.98% O_2_, 21.33% CO_2_, 58.45% N_2_, 12.54% H_2_O, and 0.7% Ar	Cement Plant (Theoretical)	EI comparison/Three-stage membrane, MDEA absorption, calcium looping	1 ton of cement production	Cradle-to-gate	Literature/Literature	GaBi/ReCipe	[32]
Polysulfone/polyvinyl amine	Spiral-wound membrane	O_2_, CO_2_, N_2_, H_2_O, and Ar. Percentages Na	Steel Mill (Theoretical)	EI comparison/Two-stage membrane, MDEA and NaOH absorptions	1 ton of steel production	Cradle-to-gate	Literature/Literature, GaBi	GaBi/ReCipe	[33]
Polyvinyl imidazole	Na	Na	Polymer manufacture (Experimental)	EI evaluation/polymer synthesis for PCC	Na	Cradle-to-gate	Experimental/Ecoinvent	SimaPro/CML	[5]
Pebax 1657	Na	10.3% O_2_, 8.5% CO_2_, 74.1% N_2_, 7.1% H_2_O	Coal-fired Power Plant (Theoretical)	EI comparison/Two-stage membrane, MEA absorption, adsorption via activated carbon	1 ton of product A and 3.5 ton of product B	Gate-to-gate	Literature/Ecoinvent	GaBi/Ecoinvent	[34]
Several polymers focusing MEEP and Pebax	Na	Varied CO_2_/N_2_: 50/50, 20/80, and 99/1	Conventional capture process (Theoretical)	EI evaluation for production/Single-stage, two-stage, and three-stage membranes	1 kg of CO_2_ capture	Gate-to-gate	GREET, literature/GREET	GREET/GREET,	[35]
Polyvinyl amine/Polyphenylene oxide	Hollow-fiber membrane	Na	Coal-fired Power Plant (Theoretical)	EI evaluation/Biomass co-firing in two-stage membrane	1 kWh of electricity generation	Cradle-to-grave	Literature/Ecoinvent	OpenLCA/CML, ReCipe	[36]
Na	Na	Na	Coal-fired Power Plant (Experimental and Theoretical)	EI comparison/Three-stage membrane, MEA absorption	Na	Not clear; production and setup	Experimental, Literature/Ecoinvent	GaBi/GaBi	[3]
Na	Na	Na	Coal-fired Power Plant (Theoretical)	EI comparison/Two-stage membrane, MEA and ammonia absorptions, calcium looping	1 kWh of electricity generation	Cradle-to-gate	Literature, industry reports/Ecoinvent, Managed LCA Content	GaBi/CML	[39]
Polyvinylidene, Polysulfone, Cellulose acetate	Hollow fiber membrane	Na	Membrane production plant (Experimental)	EI evaluation/Seven different polymer-solvent production for PCC	1000 m^2^ of membrane production	Cradle-to-gate	Experimental/Literature, Ecoinvent	SimaPro/ReCipe	[41]
Na	Na	24% CO_2_, 51% N_2_, 22% CO, and 3% H_2_	Steel Mill with CCU (Theoretical)	EI comparison/Two-stage membrane, MEA absorption, adsorption via activated carbon	1 ton of steel production	Cradle-to-gate	Literature/Literature	OpenLCA/Na	[42]

^1^ Percentages in volume. ^2^ Na refers to “not available”.

**Table 5:** Overview of reviewed studies on polymeric membrane gas absorption for PCC systems, detailing the plant type, LCA goals, functional units, system boundaries, data sources, and LCA tools and methods.

Contactor Polymer(s) Studied	Configuration	Feed/Flue Gas Composition ^1^	Plant (Approach)	Goal/PCC Systems Studied	Functional Unit	System Boundary	Foreground/Background Data Sources	LCA Tool/EIA Method	Ref.
Polysulfone ^2^/PolyActive^TM^	Hollow-fiber membrane	12.46% CO_2_ and 87.54% N_2_	Coal-fired Power Plant (Theoretical)	EI comparison/Membrane gas absorption, two-stage membrane, MEA absorption	1 ton of CO_2_ capture	Gate-to-gate	Literature, industry reports, open data sets/Ecoinvent	SimaPro/ReCipe	[2]
Polypropylene	Hollow-fiber membrane	14.1% CO_2_, 1.98% O_2_, and 83.92% N_2_	Coal-fired Power Plant (Experimental)	EI evaluation of various operational conditions/Membrane gas absorption	1 kg of CO_2_ capture	Gate-to-gate	Experimental/Ecoinvent	SimaPro/ReCipe	[1]
Polypropylene	Hollow-fiber membrane	13.53 CO_2_	Coal-fired Power Plant (Theoretical)	EI evaluation of operational scenarios/solar-assisted membrane gas absorption	1 kWh of electricity generation	Cradle-to-gate	Literature/ Ecoinvent	SimaPro/CML	[40]

^1^ Percentages in volume. ^2^ Li et al. [2] evaluated both gas separation and membrane absorption systems. While the contactor material used in the absorption configuration was not specified, polysulfone and PolyActive™ were reported as selective membrane materials in the gas separation case.

## Data Availability

The data that supports the findings of this study are available on request from the corresponding author.

## Supplementary Materials

The following supporting information can be downloaded at: https://www.mdpi.com/article/10.3390/polym18070868/s1, Figure S1: Distribution of studies by (a) research approach, (b) plant type, (c) membrane system, and (d) polymer material. Percentages are calculated within each category. In (c) and (d), some studies appear in multiple categories due to the use of more than one membrane system or polymer material; Table S1: The search results corresponding to the search strings and databases. The search strings were applied to the whole article domain. Only articles written in English were searched; Table S2: The list of included studies in the systematic review work, along with the corresponding identification methods; Table S3: Content categories for data extraction based on the research scope; Table S4: PRISMA 2020 [26] for Abstracts checklist, detailing the reporting of essential methodological, result-based, and administrative elements within the study abstract; Table S5: PRISMA 2020 item checklist [26] for systematic reviews, providing the specific locations (sections and paragraphs) within the manuscript where each reporting requirement is addressed.
