# Supplementary material for: Are Polymeric Membranes Truly Sustainable? Life Cycle Assessment Studies of Polymeric Membranes in Post-Combustion CO2 Capture: A Systematic Review
Source: Polymers (Basel). 2026 Apr 1;18(7):868. doi: 10.3390/polym18070868 (PMC13074247; doi:10.3390/polym18070868)
Supplement: Supplementary file 1 [file polymers-18-00868-s001.zip › polymers-4233300-supplementary.pdf]

Supplemental Material for *Are Polymeric Membranes Truly Sustainable? Life Cycle Assessment Studies of Polymeric Membranes in Post-Combustion CO_2 Capture: A Systematic Review*

Table S1. The search results corresponding to the search strings and databases. The search strings were applied to the whole article domain. Only articles written in English were searched.

Database	Search Strings	# of Articles	Date of Access
Google Scholar	"polymeric membrane" OR "polyvinyl imidazole" OR “polypropylene” OR "mixed matrix membrane" AND "life cycle assessment" OR "LCA" OR "life cycle analysis" OR "environmental impact assessment" OR "life cycle inventory" OR "life cycle impact assessment" AND "post-combustion CO_2 capture" OR "post-combustion carbon capture" OR "CO_2 separation" OR "carbon dioxide capture" OR "flue gas CO_2 capture" OR "post-combustion CCS" OR "CO_2 membrane separation"	387	03.01.2026
ScienceDirect	("polymeric membrane*" OR "polyvinyl imidazole" OR “polypropylene” OR "mixed matrix membrane") AND ("life cycle assessment" OR "LCA" OR "life cycle analysis" OR "environmental impact assessment" OR "life cycle inventory" OR "life cycle impact assessment") AND ("post-combustion CO_2 capture" OR "post-combustion carbon capture" OR "CO_2 separation" OR "carbon dioxide capture" OR "flue gas CO_2 capture" OR "post-combustion CCS" OR "CO_2 membrane separation")	333	04.01.2026
Web of Science	("polymeric membrane*" OR "polyvinyl imidazole" OR “polypropylene” OR "mixed matrix membrane") AND ("life cycle assessment" OR "LCA" OR "life cycle analysis" OR "environmental impact assessment" OR "life cycle inventory" OR "life cycle impact assessment") AND ("post-combustion CO_2 capture" OR "post-combustion carbon capture" OR "CO_2 separation" OR "carbon dioxide capture" OR "flue gas CO_2 capture" OR "post-combustion CCS" OR "CO_2 membrane separation")	148	05.01.2026
MDPI[1]	("Life Cycle Assessment" OR LCA) AND Polymer AND (CO2 OR Carbon)	99	05.01.2026

[1]A high-recall search string was applied to MDPI to account for terminology variations. This broader approach was feasible due to the database's contained size, yielding a manageable dataset for manual screening.

Table S2. The list of included studies in the systematic review work, along with the corresponding identification methods

No	Study Name	How study was identified	Reference
**1**	Life cycle assessment of post-combustion CO2 capture and recovery by hydrophobic polypropylene cross-flow hollow fiber membrane contactors with activated methyldiethanolamine	By the application of search strings	Akan et al., 2023
**2**	Life cycle assessment of post-combustion carbon capture and storage for the ultra-supercritical pulverized coal power plant. Science of the Total Environment, 927, 172047. https://doi.org/10.1016/j.scitotenv.2024.172047	By the application of snowballing technique	Cao et al., 2024
**3**	Environmental Impact Improvement of Chitosan-Based Mixed-Matrix Membranes Manufacture for CO2 Gas Separation by Life Cycle Assessment	By the application of search strings	Echarri et al., 2023

Table S2 *(Continued)*

No	Study Name	How study was identified	Reference
**4**	Environmental impact assessment of post-combustion CO_2 capture technologies applied to cement production plants	By the application of search strings	Galusnyak et al., 2022
**5**	Life cycle assessment of post-combustion CO_2 capture: A comparison between membrane separation and chemical absorption processes	By the application of search strings	Giordano et al., 2018
**6**	Comparative life cycle assessment of polymeric membranes: Polyacrylonitrile, polyvinylimidazole and poly (acrylonitrile-co-vinylimidazole) applied for CO_2 sequestration	By the application of search strings	Khaki et al., 2021
**7**	Environmental Impact Evaluation of CO_2 Absorption and Desorption Enhancement by Membrane Gas Absorption: A Life Cycle Assessment Study	By the application of search strings	Li et al., 2024
**8**	Membrane technology applied to steel production: Investigation based on process modelling and environmental tools	By the application of search strings	Luca and Petrescu, 2021
**9**	Carbon dioxide capture in the iron and steel industry: thermodynamic analysis, process simulation, and life cycle assessment	By the application of snowballing technique	Mio et al., 2022
**10**	Environmental performances of various CCU options in the framework of an integrated chemical plant	By the application of snowballing technique	Mirgaux et al., 2021
**11**	Life Cycle Assessment of Innovative Carbon Dioxide Selective Membranes from Low Carbon Emission Sources: A Comparative Study	By the application of search strings	Nilkar et al., 2023
**12**	China's coal power decarbonization via CO_2 capture and storage and biomass co-firing: A LCA case study in Inner Mongolia	By the application of snowballing technique	Sammarchi et al., 2022
**13**	Screening life cycle analysis of post combustion CO_2-capture technologies–a comparison of construction phase results	By the application of search strings	Troy and Wagner, 2011
**14**	Life cycle assessment of membrane-based carbon capture and storage	By the application of search strings	Troy et al., 2016
**15**	Higher efficiency and lower environmental impact of membrane separation for carbon dioxide capture in coal power plants	By the application of search strings	Wang et al., 2023
**16**	Life cycle environmental impact and economic analysis of post-combustion carbon capture technologies in supercritical coal-fired power plants	By the application of snowballing technique	Wang et al., 2025a
**17**	Assessment of the environmental impact of polymeric membrane production	By the application of search strings	Yadav et al., 2021
**18**	Life Cycle Assessment and Environmental Impact Evaluation of CCU Technology Schemes in Steel Plants	By the application of search strings	Yu et al., 2024
**19**	Post-combustion carbon capture technologies: Energetic analysis and life cycle assessment	By the application of search strings	Zhang et al., 2014
**20**	Life-cycle performance of hydrogen production via indirect biomass gasification with CO_2 capture	By the application of search strings	Susmozas et al., 2016

Table S2 *(Continued)*

No	Study Name	How study was identified	Reference
**21**	Life Cycle Assessment of Solar-Assisted Post-Combustion CO2 Capture Using Hollow Fiber Membrane Contactors	By the application of search strings	Wang et al., 2025b

Table S3. Content categories for data extraction based on the research scope

Category	Content	Rationale for extraction
General information	The authors, the year of publication, the name of the journal, etc.	To record reference information
Gap	The problem that the article aimed to address	To identify commonly addressed problems
Background information	Metadata regarding the global warming, carbon capture processes, membrane separation, and life cycle assessment	To strengthen the literature review of the current study, particularly in the introduction
Material and methods	Plant/s studied	To identify common trends
	Membrane type/s, material/s, and configuration/s used	To identify common trends
	Solvents used	
	Process approach	To determine whether the selected article was experimental, a simulation, or a full-scale application
	Description of gas separation/absorption processes	To identify different applications
	LCA details	To identify the LCA parameter choices of the reviewed studies, including the LCA goal, functional unit, system boundary, LCA tool, data source, LCA assumptions, environmental impact assessment method, and impact categories
	Additional methods	To analyze whether any hybrid approaches were used in the reviewed studies, such as the combination of LCA and techno-economic analysis
Results and discussion	Process contribution to environmental impact categories	To determine which processes in the process chain contribute the most to global warming
	Environmental impacts of polymeric membrane separation	To analyze the environmental impact of using polymeric membranes for post-combustion CO_2 capture, both qualitatively and quantitatively
	Environmental impacts of other post-combustion CO_2 separation methods	To compare other methods with polymeric membrane separation
	Sensitivity and uncertainty aspects	To determine how the reviewed studies approached the sensitivity and uncertainty of their findings
	Other findings	To discuss other findings within the research scope if needed
Conclusion	Implications	To determine the implication of the reviewed study
	Future recommendations	To identify available gaps on the subject
	Snowballing references	To extend the selected literature

Table S4. PRISMA 2020 (Page et al., 2021) for Abstracts checklist, detailing the reporting of essential methodological, result-based, and administrative elements within the study abstract.

Section and Topic	Item #	Checklist item	Reported (Yes/No)
**TITLE**			
Title	1	Identify the report as a systematic review.	Yes
**BACKGROUND**			
Objectives	2	Provide an explicit statement of the main objective(s) or question(s) the review addresses.	Yes
**METHODS**			
Eligibility criteria	3	Specify the inclusion and exclusion criteria for the review.	Yes
Information sources	4	Specify the information sources (e.g. databases, registers) used to identify studies and the date when each was last searched.	Yes
Risk of bias	5	Specify the methods used to assess risk of bias in the included studies.	Yes
Synthesis of results	6	Specify the methods used to present and synthesise results.	Yes
**RESULTS**			
Included studies	7	Give the total number of included studies and participants and summarise relevant characteristics of studies.	Yes
Synthesis of results	8	Present results for main outcomes, preferably indicating the number of included studies and participants for each. If meta-analysis was done, report the summary estimate and confidence/credible interval. If comparing groups, indicate the direction of the effect (i.e. which group is favoured).	Yes
**DISCUSSION**			
Limitations of evidence	9	Provide a brief summary of the limitations of the evidence included in the review (e.g. study risk of bias, inconsistency and imprecision).	Yes
Interpretation	10	Provide a general interpretation of the results and important implications.	Yes
**OTHER**			
Funding	11	Specify the primary source of funding for the review.	No
Registration	12	Provide the register name and registration number.	No

Table S5. PRISMA 2020 item checklist (Page et al., 2021) for systematic reviews, providing the specific locations (sections and paragraphs) within the manuscript where each reporting requirement is addressed.

Section and Topic	Item #	Checklist item	Location where item is reported
**TITLE**			
Title	1	Identify the report as a systematic review.	**Title**
**ABSTRACT**			
Abstract	2	See the PRISMA 2020 for Abstracts checklist.	**Abstract**
**INTRODUCTION**			
Rationale	3	Describe the rationale for the review in the context of existing knowledge.	**Introduction**, 3rd paragraph
Objectives	4	Provide an explicit statement of the objective(s) or question(s) the review addresses.	**Introduction:** 4rd paragraph, and **2.1. Protocol:** 1st paragraph
**METHODS**			
Eligibility criteria	5	Specify the inclusion and exclusion criteria for the review and how studies were grouped for the syntheses.	**2.3. Appraisal:** 1st paragraph
Information sources	6	Specify all databases, registers, websites, organisations, reference lists and other sources searched or consulted to identify studies. Specify the date when each source was last searched or consulted.	Supplemental table S1
Search strategy	7	Present the full search strategies for all databases, registers and websites, including any filters and limits used.	**2.2. Search:** whole subsection, and Supplemental table S1
Selection process	8	Specify the methods used to decide whether a study met the inclusion criteria of the review, including how many reviewers screened each record and each report retrieved, whether they worked independently, and if applicable, details of automation tools used in the process.	**2.4. Synthesis:** whole subsection
Data collection process	9	Specify the methods used to collect data from reports, including how many reviewers collected data from each report, whether they worked independently, any processes for obtaining or confirming data from study investigators, and if applicable, details of automation tools used in the process.	**2.4. Synthesis:** whole subsection, and Supplemental table S3
Data items	10a	List and define all outcomes for which data were sought. Specify whether all results that were compatible with each outcome domain in each study were sought (e.g. for all measures, time points, analyses), and if not, the methods used to decide which results to collect.	Supplemental table S3
	10b	List and define all other variables for which data were sought (e.g. participant and intervention characteristics, funding sources). Describe any assumptions made about any missing or unclear information.	Table 4 and 5
Study risk of bias assessment	11	Specify the methods used to assess risk of bias in the included studies, including details of the tool(s) used, how many reviewers assessed each study and whether they worked	**2.3.1. Quality Assessment and Risk of Bias:** whole subsection

Section and Topic	Item #	Checklist item	Location where item is reported
		independently, and if applicable, details of automation tools used in the process.	
Effect measures	12	Specify for each outcome the effect measure(s) (e.g. risk ratio, mean difference) used in the synthesis or presentation of results.	**2.5. Analysis and Reporting:** 2nd paragraph
Synthesis methods	13a	Describe the processes used to decide which studies were eligible for each synthesis (e.g. tabulating the study intervention characteristics and comparing against the planned groups for each synthesis (item #5)).	**2.4. Synthesis:** whole subsection, and Supplemental table S3
	13b	Describe any methods required to prepare the data for presentation or synthesis, such as handling of missing summary statistics, or data conversions.	**2.5. Analysis and Reporting:** 2nd paragraph
	13c	Describe any methods used to tabulate or visually display results of individual studies and syntheses.	**2.5. Analysis and Reporting:** 1st paragraph, Figure 2
	13d	Describe any methods used to synthesize results and provide a rationale for the choice(s). If meta-analysis was performed, describe the model(s), method(s) to identify the presence and extent of statistical heterogeneity, and software package(s) used.	Supplemental table S3
	13e	Describe any methods used to explore possible causes of heterogeneity among study results (e.g. subgroup analysis, meta-regression).	**3.3.3. Life Cycle Impact Assessment and Interpretation:** whole subsection
	13f	Describe any sensitivity analyses conducted to assess robustness of the synthesized results.	**3.4.4. Sensitivity and Robustness Assessment of Comparative Environmental Impact Results:** 1st paragraph
Reporting bias assessment	14	Describe any methods used to assess risk of bias due to missing results in a synthesis (arising from reporting biases).	**2.3.1. Quality Assessment and Risk of Bias:** whole subsection, and **3.1. Study Quality and Reporting Transparency:** whole subsection
Certainty assessment	15	Describe any methods used to assess certainty (or confidence) in the body of evidence for an outcome.	**3.4.5. Evaluating the Quality and Comparability of LCA Evidence:** qualitatively discussed in the 2th and 5th paragraphs
**RESULTS**			
Study selection	16a	Describe the results of the search and selection process, from the number of records identified in the search to the number of studies included in the review, ideally using a flow diagram.	**2.2. Search:** 2nd paragraph, and Figure 1
	16b	Cite studies that might appear to meet the inclusion criteria, but which were excluded, and explain why they were excluded.	Not Applicable
Study characteristics	17	Cite each included study and present its characteristics.	Table 4 & 5, Supplemental Table S2
Risk of bias in studies	18	Present assessments of risk of bias for each included study.	Figure 3
Results of individual studies	19	For all outcomes, present, for each study: (a) summary statistics for each group (where appropriate) and (b) an effect estimate and its precision (e.g. confidence/credible interval), ideally using structured tables or plots.	Figure 5 & 6

Section and Topic	Item #	Checklist item	Location where item is reported
Results of syntheses	20a	For each synthesis, briefly summarise the characteristics and risk of bias among contributing studies.	Figure 3, Table 4 & 5
	20b	Present results of all statistical syntheses conducted. If meta-analysis was done, present for each the summary estimate and its precision (e.g. confidence/credible interval) and measures of statistical heterogeneity. If comparing groups, describe the direction of the effect.	Figure 2, 5 & 6
	20c	Present results of all investigations of possible causes of heterogeneity among study results.	**3.4.5. Evaluating the Quality and Comparability of LCA Evidence:** whole section
	20d	Present results of all sensitivity analyses conducted to assess the robustness of the synthesized results.	**3.4.4. Sensitivity and Robustness Assessment of Comparative Environmental Impact Results:** 2nd and 3rd paragraphs
Reporting biases	21	Present assessments of risk of bias due to missing results (arising from reporting biases) for each synthesis assessed.	**3.1. Study Quality and Reporting Transparency:** whole subsection, and Figure 3
Certainty of evidence	22	Present assessments of certainty (or confidence) in the body of evidence for each outcome assessed.	**3.4.5. Evaluating the Quality and Comparability of LCA Evidence:** qualitatively discussed in the 2th , 3rd, and 5th paragraphs
**DISCUSSION**			
Discussion	23a	Provide a general interpretation of the results in the context of other evidence.	**3.2. Polymeric Membranes in Post-Combustion CO_2 Capture**, and **3.3. Life Cycle Assessment of Polymeric Membranes for Post-Combustion CO_2 Capture:** whole sections combine R&D of evidence
	23b	Discuss any limitations of the evidence included in the review.	**3.4.5. Evaluating the Quality and Comparability of LCA Evidence:** whole section
	23c	Discuss any limitations of the review processes used.	**4. Limitations**: whole section
	23d	Discuss implications of the results for practice, policy, and future research.	**5. Recommendations and Future Research Directions** and **6. Conclusion:** whole sections
**OTHER INFORMATION**			
Registration and protocol	24a	Provide registration information for the review, including register name and registration number, or state that the review was not registered.	The review was not registered.
	24b	Indicate where the review protocol can be accessed, or state that a protocol was not prepared.	**2.1. Protocol** section describes the review protocol
	24c	Describe and explain any amendments to information provided at registration or in the protocol.	Not Applicable
Support	25	Describe sources of financial or non-financial support for the review, and the role of the funders or sponsors in the review.	Acknowledgments statement is presented in the study

Section and Topic	Item #	Checklist item	Location where item is reported
Competing interests	26	Declare any competing interests of review authors.	Conflicts of Interest statement is presented in the study
Availability of data, code and other materials	27	Report which of the following are publicly available and where they can be found: template data collection forms; data extracted from included studies; data used for all analyses; analytic code; any other materials used in the review.	Data Availability Statement is presented in the study

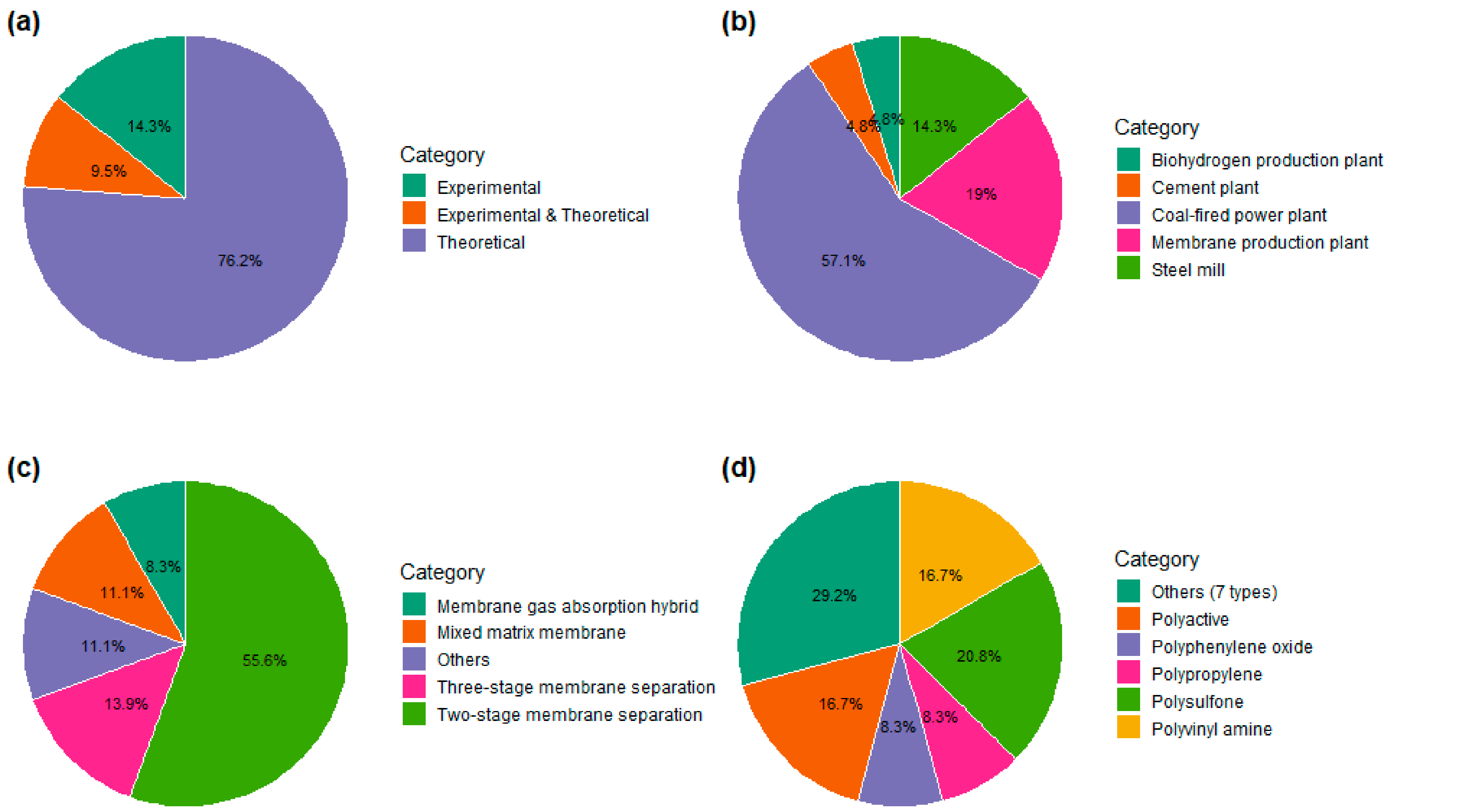

Figure S1. Distribution of studies by (a) research approach, (b) plant type, (c) membrane system, and (d) polymer material. Percentages are calculated within each category. In (c) and (d), some studies appear in multiple categories due to the use of more than one membrane system or polymer material.

References

Akan, A. P., Chau, J., Gullu, G., & Sirkar, K. K. (2023). Life cycle assessment of post-combustion CO_2 capture and recovery by hydrophobic polypropylene cross-flow hollow fiber membrane contactors with activated methyldiethanolamine. *Atmosphere*, 14(3), 490. https://doi.org/10.3390/atmos14030490

Cao, X., Chen, S., & Xiang, W. (2024). Life cycle assessment of post-combustion carbon capture and storage for the ultra-supercritical pulverized coal power plant. *Science of the Total Environment*, 927, 172047. https://doi.org/10.1016/j.scitotenv.2024.172047

Echarri, I., Casado-Coterillo, C., Rumayor, M., Navajas, A., & Gandía, L. M. (2023). Environmental Impact Improvement of Chitosan-Based Mixed-Matrix Membranes Manufacture for CO_2 Gas Separation by Life Cycle Assessment. *Chemical Engineering & Technology*, 46(10), 2184-2191. https://doi.org/10.1002/ceat.202200397

Galusnyak, S. C., Petrescu, L., & Cormos, C. C. (2022). Environmental impact assessment of post-combustion CO_2 capture technologies applied to cement production plants. *Journal of Environmental Management*, 320, 115908. https://doi.org/10.1016/j.jenvman.2022.115908

Giordano, L., Roizard, D., & Favre, E. (2018). Life cycle assessment of post-combustion CO_2 capture: A comparison between membrane separation and chemical absorption processes. *International Journal of Greenhouse Gas Control*, 68, 146-163. https://doi.org/10.1016/j.ijggc.2017.11.008

Khaki, E., Abyar, H., Nowrouzi, M., Younesi, H., Abdollahi, M., & Enderati, M. G. (2021). Comparative life cycle assessment of polymeric membranes: Polyacrylonitrile, polyvinylimidazole and poly (acrylonitrile-co-vinylimidazole) applied for CO_2 sequestration. *Environmental Technology & Innovation*, 22, 101507. https://doi.org/10.1016/j.eti.2021.101507

Li, F., Lv, Y., Bi, J., Zhang, H., Zhao, W., Su, Y., Du, T., & Mu, J. (2024). Environmental Impact Evaluation of CO_2 Absorption and Desorption Enhancement by Membrane Gas Absorption: A Life Cycle Assessment Study. *Energies*, 17(10), 2371. https://doi.org/10.3390/en17102371

Luca, A. V., & Petrescu, L. (2021). Membrane technology applied to steel production: Investigation based on process modelling and environmental tools. *Journal of Cleaner Production*, 294, 126256. https://doi.org/10.1016/j.jclepro.2021.126256

Mio, A., Petrescu, L., Luca, A. V., Galusnyak, S. C., Fermeglia, M., & Cormos, C. C. (2022). Carbon dioxide capture in the iron and steel industry: thermodynamic analysis, process simulation, and life cycle assessment. *Chemical and Biochemical Engineering Quarterly*, 36(4), 255-271. https://doi.org/10.15255/CABEQ.2022.2123

Mirgaux, O., Anselmi, H., & Patisson, F. (2021). Environmental performances of various CCU options in the framework of an integrated chemical plant. *Membranes*, 11(11), 815. https://doi.org/10.1016/j.jenvman.2022.115908

Nilkar, A. S., Orme, C. J., Klaehn, J. R., Zhao, H., & Adhikari, B. (2023). Life Cycle Assessment of Innovative Carbon Dioxide Selective Membranes from Low Carbon Emission Sources: A Comparative Study. *Membranes*, 13(4), 410. https://doi.org/10.3390/membranes13040410

Page, M. J., McKenzie, J. E., Bossuyt, P. M., Boutron, I., Hoffmann, T. C., Mulrow, C. D., ... & Moher, D. (2021). The PRISMA 2020 statement: an updated guideline for reporting systematic reviews. *BMJ*, 372. https://doi.org/10.1136/bmj.n71

Sammarchi, S., Li, J., Izikowitz, D., Yang, Q., & Xu, D. (2022). China's coal power decarbonization via CO2 capture and storage and biomass co-firing: A LCA case study in Inner Mongolia. *Energy*, 261, 125158. https://doi.org/10.1016/j.energy.2022.125158

Susmozas, A., Iribarren, D., Zapp, P., Linβen, J., & Dufour, J. (2016). Life-cycle performance of hydrogen production via indirect biomass gasification with CO_2 capture. *International journal of hydrogen energy*, 41(42), 19484-19491. https://doi.org/10.1016/j.ijhydene.2016.02.053

Troy, S. V., & Wagner, H. J. (2011). Screening life cycle analysis of post combustion CO_2-capture technologies–a comparison of construction phase results. *Energy Procedia*, 4, 480-487. https://doi.org/10.1016/j.egypro.2011.01.078

Troy, S., Schreiber, A., & Zapp, P. (2016). Life cycle assessment of membrane-based carbon capture and storage. *Clean technologies and environmental policy*, 18(6), 1641-1654. https://doi.org/10.1007/s10098-016-1208-x

Wang, L., Zhou, H., Liu, X., Mu, J., Bi, J., Jin, Y., ... & Lv, Y. (2025b). Life Cycle Assessment of Solar-Assisted Post-Combustion CO_2 Capture Using Hollow Fiber Membrane Contactors. *Frontiers in Heat and Mass Transfer*, 23(6), 1811-1832. https://doi.org/10.32604/fhmt.2025.071222

Wang, Y., Pan, Z., Zhang, W., Huang, S., Yu, G., Soltanian, M. R., Lichtfouse, E., & Zhang, Z. (2023). Higher efficiency and lower environmental impact of membrane separation for carbon dioxide capture in coal power plants. *Environmental Chemistry Letters*, 21(4), 1951-1958. https://doi.org/10.1007/s10311-023-01596-0

Wang, Y., Shao, S., Gao, Q., Zhang, Y., Wang, X., & Gao, X. (2025a). Life cycle environmental impact and economic analysis of post-combustion carbon capture technologies in supercritical coal-fired power plants. *Environmental Impact Assessment Review*, 114, 107933. https://doi.org/10.1016/j.energy.2022.125158

Yadav, P., Ismail, N., Essalhi, M., Tysklind, M., Athanassiadis, D., & Tavajohi, N. (2021). Assessment of the environmental impact of polymeric membrane production. *Journal of Membrane Science*, 622, 118987. https://doi.org/10.1016/j.memsci.2020.118987

Yu, C., Li, Y., Wang, L., Jiang, Y., Wang, S., Du, T., & Wang, Y. (2024). Life Cycle Assessment and Environmental Impact Evaluation of CCU Technology Schemes in Steel Plants. *Sustainability*, 16(23), 10207. https://doi.org/10.3390/su162310207

Zhang, X., Singh, B., He, X., Gundersen, T., Deng, L., & Zhang, S. (2014). Post-combustion carbon capture technologies: Energetic analysis and life cycle assessment. International Journal of Greenhouse Gas Control, 27, 289-298. https://doi.org/10.1016/j.ijggc.2014.06.016
